# The contribution of HIV point-of-care tests in early HIV diagnosis: community-based HIV testing monitoring in Catalonia, 1995 to 2018

**DOI:** 10.2807/1560-7917.ES.2020.25.43.1900424

**Published:** 2020-10-29

**Authors:** Laura Fernàndez-López, Juliana Reyes-Urueña, Anna Conway, Jorge Saz, Adriana Morales, Jaime Quezadas, Jordi Baroja, Anna Rafel, Ander Pazos, Anna Avellaneda, Mercè Meroño, Lorena Andreo, Lluís Romero, Anna Lara, Araceli Otón, Benet Rifà, Rosa Mansilla, Joan Colom, Jordi Casabona

**Affiliations:** 1 Centre for Epidemiological Studies on Sexually Transmitted Infections and HIV/AIDS of Catalonia (CEEISCAT), Catalan Health Department, Barcelona, Spain; 2 Centros de Investigación Biomédica en Red Epidemiología y Salud Pública (CIBERESP), Spain; 3 The Institute for Health Science Research Germans Trias i Pujol (IGTP), Badalona, Spain; 4 BCN Checkpoint, Barcelona, Spain; 5 Stop Sida, Barcelona, Spain; 6 Associació Ciutadana Antisida de Catalunya (ACASC), Barcelona, Spain; 7 Centre Jove d’Atenció a les Sexualitats (CJAS), Barcelona, Spain; 8 Associació Antisida de Lleida, Lleida, Spain; 9 Gais Positius, Barcelona, Spain; 10 Actuavallès, Sabadell, Spain; 11 Fundació Àmbit Prevenció, Barcelona, Spain; 12 CAS/ARD Lluís Companys, Creu Roja Barcelona, Barcelona, Spain; 13 Assexora’Tgn (Associacio Comunitària de Salut Sexual del Camp de Tarragona), Tarragona, Spain; 14 Associació Comunitària Anti Sida de Girona (ACAS), Girona, Spain; 15 Creu Roja Tarragona, Tarragona, Spain; 16 Section for Surveillance, Prevention and Control of Sexually Transmitted Infections and HIV, Public Health Agency of Catalonia, Barcelona, Spain; 17 Programme for Prevention, Control and Treatment of HIV, STIs and Viral Hepatitis, Public Health Agency of Catalonia, Barcelona, Spain; 18 Department of Paediatrics, Obstetrics and Gynaecology, and Preventive Medicine, Universitat Autònoma de Barcelona, Bellaterra, Spain

**Keywords:** HIV infections, diagnosis, prevention and control, early diagnosis, Community Health Services, statistics and numerical data, testing, sentinel surveillance

## Abstract

**Background:**

Community-based HIV testing services combined with the use of point-of-care tests (POCT) have the potential to improve early diagnosis through increasing availability, accessibility and uptake of HIV testing.

**Aim:**

To describe community-based HIV testing activity in Catalonia, Spain, from 1995 to 2018, and to evaluate the impact of HIV POCT on the HIV continuum of care.

**Methods:**

A community-based network of voluntary counselling and testing services in Catalonia, Spain has been collecting systematic data on activity, process and results since 1995. A descriptive analysis was performed on pooled data, describing the data in terms of people tested and reactive screening test results.

**Results:**

Between 1995 and 2018, 125,876 HIV tests were performed (2.1% reactive). Since the introduction of HIV POCT in 2007, a large increase in the number of tests performed was observed, reaching 14,537 tests alone in 2018 (1.3% reactive). Men who have sex with men (MSM), as a proportion of all people tested, has increased greatly over time reaching 74.7% in 2018. The highest percentage of reactive tests was found in people who inject drugs followed by MSM. The contribution of community-based HIV testing to the overall total notified cases in the Catalonia HIV registry has gradually increased, reaching 37.9% in 2018, and 70% of all MSM cases. In 2018, the percentage of individuals with a reactive screening test who were linked to care was 89.0%.

**Conclusion:**

Our study reinforces the important role that community-based HIV POCT has on the diagnosis of HIV in key populations.

## Introduction

In recent years, efforts to reach the 90–90–90 targets (90% of all people living with HIV knowing their HIV status, 90% of all people diagnosed with HIV receiving antiretroviral therapy and 90% of all people receiving antiretroviral therapy having viral suppression) advocated by the Joint United Nations Programme on HIV/AIDS (UNAIDS), have led to an improvement in accessibility and coverage of testing programmes. This, in turn, has reduced the number of people living with undiagnosed HIV infection and increased early diagnoses [1]. Monitoring and evaluation (M&E) is an essential component of any effective testing programme. While strategic information should guide the design of testing initiatives, M&E permits continuous evaluation of targets and programme effectiveness, efficiency and impact. Such data can prove invaluable in planning improvements [2].

Catalonia is an autonomous community located in the north-east of Spain. In 2018, it had a population of 7,543,825 inhabitants. This region has a low-level HIV epidemic, where high levels of infection are found only in specific groups, particularly men who have sex with men (MSM). As of 31 December 2017, Catalonia had a rate of 8.1 HIV diagnoses per 100,000 inhabitants, with 53.6% of all diagnoses in 2017 attributed to MSM [3].

In Catalonia, HIV testing M&E forms part of the Integrated AIDS/HIV/STI Surveillance System of Catalonia (SIVES) [4] and is based on two main sources of information: (i) the network of public hospital laboratories, primary healthcare centre laboratories and private laboratories (HIVLABCAT), which have voluntarily reported diagnostic HIV testing and results since 1992; and (ii) the network of community-based voluntary counselling and testing (CBVCT) services, which has offered free, anonymous, voluntary and confidential counselling and testing since 1995 [5]. This report will focus on the data collected by the CBVCT network.

CBVCT services are considered an effective strategy for HIV testing, especially for key populations [6,7], and have expanded in the European Union/European Economic Area (EU/EEA) since 2010 through a variety of service delivery models [8]. This strategy has been proven to increase the availability, accessibility and uptake of HIV testing in order to reduce the number of people who do not know their HIV status or who are diagnosed late [9], impacting the first 90 target set by UNAIDS [10]. In addition, this strategy increases the proportion of first-time testers, increases the proportion of participants who undertook follow-up CD4 tests after diagnosis, detects patients at an earlier stage of infection, increases the number of new HIV diagnoses, and potentially reduces the stigma and discrimination faced by key populations [6].

A systematic review found that the use of HIV point-of-care tests (POCT) as part of CBVCT interventions, combined with behavioural interventions either at individual or community level, has the potential for enormous impact on the HIV epidemic [11]. Scaling up the CBVCT service model was thought to increase the likelihood of achieving the 90–90–90 target by 2020 [12], but the scale up in Europe has been impacted by limited funding, poor integration with national HIV programmes and regulatory barriers. There is a need for guidance to address these implementation challenges, including M&E, and a need to assist countries in developing, implementing and evaluating national policies [13].

Community-based testing started in Catalonia with only a few sites offering traditional testing, where a nurse was required to perform venepuncture and send the blood sample to a laboratory. Traditional testing was replaced with HIV POCT in 2007, which allowed the expansion of testing programmes in the community. Since 2007, more sites have been offering HIV POCT, and the number of tests performed has increased exponentially [5].

Catalonia has experience in the scaling up of CBVCT interventions using HIV POCT with linkage to care, support and treatment services, within a solid M&E framework. Here, our aims are to describe HIV testing activity among those CBVCT services participating in the DEVO (an abbreviation of ‘voluntary detection’ in Catalan) network from 1995 to 2018 in order to evaluate HIV POCT contribution in the HIV continuum of care.

## Methods

### Settings

In 1995, the Catalan Health Department (currently, the Public Health Agency of Catalonia, ASPCAT) funded a network of CBVCT services to offer free, voluntary and confidential HIV testing in the region. The purpose of the DEVO network was to complement existing facility-based HIV testing. The DEVO network has since expanded from four CBVCT services in 1995 to the current 12 (becoming six organizations in 2001, seven in 2003, eight in 2004, 10 in 2008 and 12 in 2010), mainly operated by NGOs and serving the general population or, in some cases, key populations: MSM, sex workers (SW), young people (under 30 years old), and people who inject drugs (PWID). The participating organisations of the network are all CBVCT services providing HIV testing by trained lay providers through community and outreach services. In addition to providing HIV testing, most organisations perform syphilis and hepatitis C testing and additional HIV prevention activities. One of the organisations also offers other STI tests. Peers and other lay providers have been trained to perform and interpret rapid diagnostic tests with finger-prick blood samples.

Every person who receives a preliminary reactive test is referred to a laboratory or to an infectious disease specialist who conducts a confirmatory test. The diagnosed clients are then linked with appropriate specialist services.

### Data collection

The DEVO network has been collecting systematically standardized data on activity, process and results since 1995. Since 2014, the DEVO network has formed part of the community-based testing (COBATEST) network, a European network of CBVCT services based on the DEVO network experience [8,14]. Since then, all except one of the CBVCT services use the COBATEST data collection tool (which is based on the DEVO data collection tool) and COBATEST web-based data entry tool through which data can be extracted and analysed in collaboration with the Centre for Epidemiological Studies on Sexually Transmitted Infections and HIV/AIDS of Catalonia (CEEISCAT) as part of the Public Health Agency of Catalonia (ASPCAT). One of the CBVCT services uses their own data collection tool, and shares the minimum agreed data with CEEISCAT. For monitoring and evaluation purposes, the network currently uses the standardised core indicators defined in the COBATEST network [15], aligned with UNAIDS, World Health Organization (WHO) and European Centre for Disease Prevention and Control (ECDC) recommendations [16-18].

Data collected in the DEVO network include basic demographic information on the tester, test location, testing history, risk behaviour and results of HIV, syphilis and hepatitis C testing. Since 2014, services in the DEVO network have used a unique identifier for each client, ensuring anonymity while allowing the identification of repeat testers and recording the correct number of individuals tested.

### Test used

From 1995 to 2007, a conventional laboratory test with phlebotomy was used, from 2007 to 2012 the Determine HIV–1/2 rapid test (Abbott Laboratories, Abbott Park, IL, United States) was used, and since 2012 the new Alere Determine HIV–1/2 Ag/Ab Combo (Abbott Laboratories) test has been used. With both POCTs, the results were obtained in 15–20 min (15 min for Determine HIV–1/2 rapid test and 20 min for Alere Determine HIV–1/2 Ag/Ab Combo), and test accuracy is very high (Determine HIV–1/2 rapid test: sensitivity 99.6% (95% CI: 99.2–99.8), specificity 99.9% (95% CI: 99.8–100.0) [19,20]; Alere Determine HIV–1/2 Ag/Ab Combo: sensitivity 99.9% (95% CI: 99.4–100.0), specificity 99.8% (95% CI: 99.5–99.9) [21].

### Data analysis

The descriptive analysis was performed on pooled data from 1995 to 2018 and included: (i) the whole time period 1995–2018; (ii) each year individually; (iii) the percentage of people tested distributed by gender, age, nationality and transmission group; (iv) the percentage of individuals with a confirmed positive test before 2007 and the percentage of individuals with a reactive screening test after 2017 distributed by gender, age, nationality and transmission group. Variables included: gender (men, women, transgender), age, nationality (foreign national defined as born in a country other than Spain, or local defined as born in Spain) and transmission group (constructed as hierarchical, mutually exclusive risk categories in the following order of priority: PWID, male sex workers (MSW), MSM, female sex workers (FSW), heterosexual women (HW), heterosexual men (HM)).

For Figures 4 and 5 the MSW group was added to the MSM group in order to present all the MSM population together.

**Figure 1:**
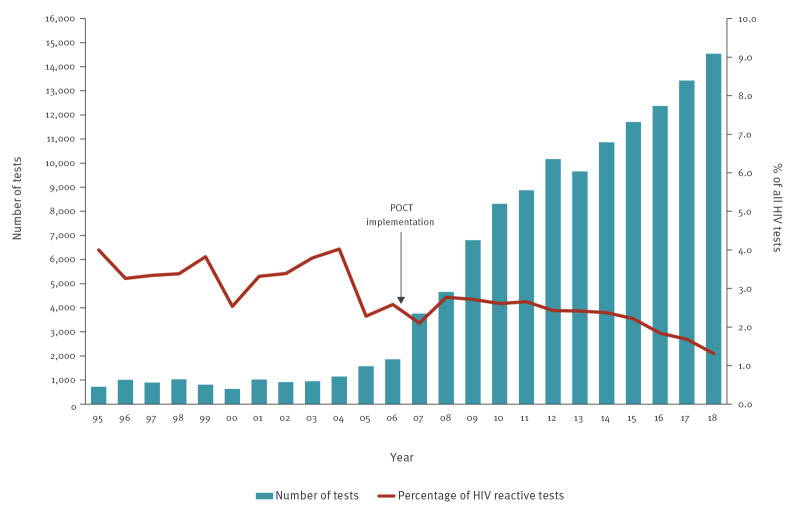
Number of HIV tests performed^a^ and percentage of reactive tests by year, Catalonia, Spain, 1995–2018 (n = 129,117)

DEVO: voluntary detection; POCT: point-of-care test.

^a^ Only HIV tests performed within the DEVO network were included.

The lines in Figure 4 chart the evolution of the percentage of reactive screening tests per year by transmission group and were smoothed using the centred moving average method. Using this method, data points were modified four times, each time the average of raw observations at a given point in time was calculated using that point, the one immediately prior and the one immediately after. This method allows for smoothing out short-term fluctuations and highlights long-term trends or cycles [22]. To test trends in Figures 1 and 4, a Pearson’s chi-squared test was used.

Linkage to care was defined as ‘entry into healthcare or follow-up by a HIV specialist or a HIV unit after a reactive or confirmatory HIV test at a community testing facility’ according the definition established in the Euro HIV EDAT project, co-funded by the European Commission [23], and all linkage to care information was collected from patient feedback.

In order to evaluate the contribution of community testing to the total number of diagnosed cases in Catalonia, data from the DEVO network and the Catalonia HIV registry were triangulated, considering that reactive tests detected in the DEVO network were linked to care and therefore were noted in the Catalonia HIV registry. The percentage of HIV cases diagnosed in the community and registered in the Catalonia HIV registry was calculated from 2001 to 2017, the period where Catalonia HIV registry data were available.

All percentages were calculated excluding missing values (which represented less than 5%). A p value below 0.001 was considered for statistical significance. Data analysis was performed using PASW Statistics for Windows, version 18.0 (SPSS Inc., Chicago, United States).

### Ethical statement

Ethical approval was not needed at the beginning of the project in 1995, as no biological samples were preserved for the study and data collected from the clients were anonymous and part of the routine services of CBVCT centres. Since the introduction of POCT, each client taking a POCT gave informed consent stating that they understood the POCT was not a diagnostic test. Since 2014 when the DEVO network joined the COBATEST network, the CBVCT services signed an agreement ensuring they fulfilled the General Data Protection Regulation, where each client has to sign an informed consent explaining the use of the data collected.

## Results

Between 1995 and 2018, 129,117 HIV tests were performed by the DEVO network, of which 2.1% were reactive. The increase in the number of tests performed by the CBVCT services by year was relatively low until 2006, ranging from 716 in 1995 to 1,849 in 2006 (Figure 1).

With the introduction of the HIV POCTs at the end of 2006, there was a 102.9% increase in the number of HIV tests performed in 2007 compared to 2006 (analysis published in a previous study [5]). In 2018, the number of HIV tests peaked at 14,537, of which 1.3% (n = 191) were reactive. From 2006 to 2018 there was an increase of 686.2% in number of tests performed (from 1,858 tests performed in 2006 to 14,537 in 2018), with an average annual increase of 21.1%. In the past 10 years, the percentage of reactive tests has been decreasing (statistically significant trend, p < 0.0001), from 2.8% (129/4,653) in 2008 to 1.3% (191/14,537) in 2018.

HIV testing activity differed greatly between CBVCT services, with one organisation, which works only with MSM, performing on average more than half of the total number of HIV tests in the DEVO network.

The Table shows the evolution by year on tests performed, people tested and number of reactive screening tests disaggregated by gender, age group, origin and transmission group.

**Table:** Number of HIV tests performed^a^, people tested and reactive tests by gender, age group, origin and transmission group, Catalonia, Spain, 1995–2018 (n = 129,117)

Year	1995	1996	1997	1998	1999	2000	2001	2002	2003	2004	2005	2006	2007	2008	2009	2010	2011	2012	2013	2014	2015	2016	2017	2018	Total
**All tests**																									
Test performed	725	1,011	899	1,034	811	631	1,025	915	949	1,144	1,576	1,858	3,757	4,653	6,806	8,317	8,875	10,168	9,654	10,867	11,705	12,371	13,432	14,537	129,117
People tested	NA	NA	NA	NA	NA	NA	NA	NA	NA	NA	NA	NA	NA	NA	NA	NA	NA	NA	NA	9,197^b^	10,536^b^	9,815^b^	10,076^b^	10,894^b^	NA
Gender (n = 112,732)^b^																									
*Men*	423	633	583	615	497	358	581	563	582	734	945	1,162	2,553	3,108	4,690	5,702	6,288	7,275	6,953	7,947	8,091	8,119	8,404	9,117	86,837
*Women*	292	372	305	417	312	273	442	350	366	403	627	691	1,111	1,420	1,709	1,946	1,772	1,741	1,528	1,436	1,536	1,528	1,524	1,563	24,135
*Transgender*	0	0	0	0	0	0	0	0	0	0	0	0	77	113	111	115	162	129	120	146	192	182	157	205	1,713
Age group (years) (n = 112,410)^b^																									
*≤ 24*	265	414	370	466	338	314	434	334	307	354	470	470	794	1,053	1,376	1,644	1,629	1,639	1,580	1,933	2,081	2,057	2,119	2,541	25,209
*25–34*	304	447	372	399	337	220	433	434	456	527	744	889	1,839	2,210	3,093	3,573	3,681	4,019	3,695	3,939	3,925	3,811	3,910	4,300	48,270
*35–44*	52	106	119	125	101	78	123	110	144	196	273	360	825	984	1,474	1,793	2,036	2,475	2,355	2,494	2,507	2,514	2,561	2,499	26,625
*≥ 45*	36	32	36	44	32	18	35	37	36	67	87	136	287	377	546	760	883	1,013	971	1,163	1,306	1,345	1,387	1,543	12,306
Origin (n = 121,087)^c^																									
*Local*	NA	NA	NA	NA	NA	NA	NA	673	667	755	1,046	1,147	2,341	3,019	4,203	4,976	5,227	5,899	5,576	6,097	6,096	5,851	5,889	6,035	66,346
*Foreign national*	NA	NA	NA	NA	NA	NA	NA	242	280	389	527	706	1,408	1,615	5,135	5,911	6,121	6,431	5,931	3,390	3,631	3,583	4,123	4,775	54,741
Transmission group (n = 106,992)																									
*PWID*	42	214	158	115	118	32	160	118	104	141	72	65	131	149	154	159	194	158	121	95	45	49	53	71	2,734
*MSW*	0	9	7	5	2	1	10	7	18	18	31	64	140	157	208	215	255	238	197	327	384	332	337	221	3,201
*MSM*	167	218	220	219	167	141	196	172	145	214	219	380	1,201	1,445	2,856	3,687	4,131	5,079	5,060	6,179	6,569	6,853	7,231	7,988	61,154
*FSW*	1	22	19	21	52	16	114	73	62	64	157	232	247	285	291	401	454	383	278	284	398	382	271	254	4,890
*HM*	260	260	218	338	231	237	267	220	245	277	404	379	897	900	1220	1,443	1,426	1,470	1,322	1,510	1,266	1,045	939	1,034	17,929
*HW*	213	250	231	302	224	177	259	220	235	286	466	466	709	989	1,159	1,292	1,070	1,100	1,042	1,134	1,130	1,136	1,243	1,132	17,084
Reactive tests																									
HIV reactive tests	29	33	30	35	31	16	34	31	36	46	36	48	79	129	185	217	236	247	233	258	259	228	226	191	2,848
Gender (n = 2,923)																									
*Men*	25	23	22	30	25	13	27	21	31	40	28	41	68	111	166	204	215	228	224	241	240	210	210	179	2,646
*Women*	4	10	8	5	6	3	7	8	8	5	8	7	9	9	11	10	9	12	6	5	4	4	4	4	169
*Transgender*	0	0	0	0	0	0	0	0	0	0	0	0	5	9	8	3	12	7	3	12	15	14	12	8	106
Age group (years) (n = 2,909)																									
*≤ 24*	5	2	4	6	5	7	10	6	4	7	8	3	10	16	21	27	32	18	34	44	34	24	27	38	394
*25–34*	13	23	18	18	17	5	19	15	17	21	16	29	31	75	88	111	105	130	97	122	108	111	114	85	1,405
*35–44*	4	4	8	8	9	3	5	8	12	14	7	14	29	30	63	55	79	62	81	69	90	61	58	44	823
*≥ 45*	3	1	0	3	0	1	0	2	3	4	5	2	9	7	12	23	20	36	21	23	27	31	27	24	287
Origin (n = 2,711)																									
*Local*	NA	NA	NA	NA	NA	NA	NA	14	15	21	18	21	30	59	93	101	114	139	120	123	138	108	83	60	1,272
*Foreign national*	NA	NA	NA	NA	NA	NA	NA	17	21	25	18	27	49	70	91	116	122	107	113	135	121	119	143	131	1,439
Transmission group (n = 2,786)																									
*PWID*	5	16	12	17	17	8	19	17	15	26	6	11	5	12	14	4	11	9	4	1	1	4	3	1	240
*MSW*	0	0	1	2	0	0	0	1	2	4	7	7	9	14	15	15	21	22	19	40	36	34	26	13	288
*MSM*	17	9	10	8	7	4	6	5	7	7	7	16	40	70	132	174	180	186	191	205	213	181	191	168	2,050
*FSW*	0	0	1	0	2	0	2	0	4	1	4	2	4	4	2	2	2	1	2	3	1	0	0	0	39
*HM*	4	3	4	3	1	1	4	2	1	0	1	3	3	3	7	6	6	9	6	2	5	4	3	5	86
*HW*	2	4	2	5	4	2	2	2	2	2	2	1	2	4	4	5	3	7	3	7	3	3	3	4	83

DEVO: voluntary detection; FSW: female sex worker; HM: heterosexual men; HW: heterosexual women; MSM: men who have sex with men; MSW: male sex worker; NA: not available; PWID: people who inject drugs.

^a^ Only HIV tests performed within the DEVO network were included.

^b^ From 2014 to 2018 the disaggregated data refer to people tested instead of number of tests performed.

^c^ Foreign national: person born in country other than Spain; local: person born in Spain.

**Figure 2:**
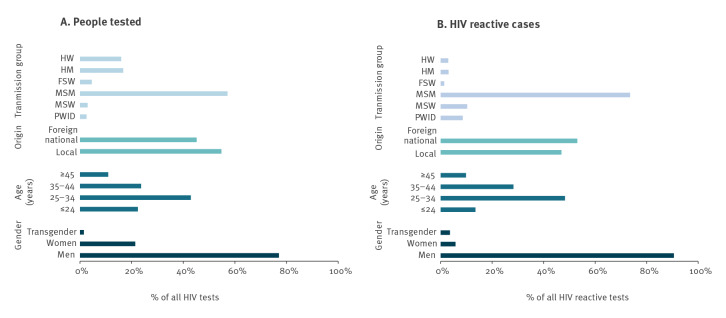
Gender, age, origin and transmission group of (A) all people tested for HIV (n = 112,732^a^) and (B) HIV reactive tests (n = 2,848), Catalonia, Spain, 1995–2018

DEVO: voluntary detection; Foreign national: person born in country other than Spain; FSW: female sex worker; HM: heterosexual men; HW: heterosexual women; local: person born in Spain; MSM: men who have sex with men; MSW: male sex worker; PWID: people who inject drugs.

^a^ Only people tested within the DEVO network were included. Before 2014 disaggregated data refers to number of tests instead of people tested.

Between 1995 and 2018, 77.0% (86,837/112,732) of the total people tested at the community sites were men, and 92.9% (2,646/2,848) of reactive tests were in men. In men and women, the age group with the most people tested and most reactive tests was 25–34 years old (Figure 2). Foreign nationals accounted for 45.2% of the total number of people tested, and 53.1% of the total number of reactive tests. MSM accounted for 57.2% of all people tested, and 73.58% of the total number of reactive tests.

**Figure 3:**
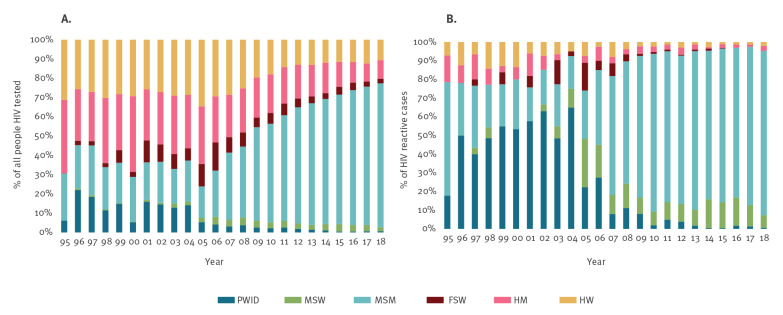
Distribution by transmission group of (A) all people HIV tested^a^ (n=106,992) and (B) HIV reactive tests (n=2,786), Catalonia, Spain, 1995–2018

DEVO: voluntary detection; FSW: female sex worker; HM: heterosexual men; HW: heterosexual women; MSM: men who have sex with men; MSW: male sex worker; PWID: people who inject drugs.

^a^ Only people tested within the DEVO network were included. Before 2014 disaggregated data refers to number of tests instead of people tested.

Figure 3 describes the contribution of each transmission group to the total number of people tested, and the total number of reactive tests between 1995 and 2018. It shows that MSM as a proportion of all people tested has increased greatly over time, reaching 74.7% (7,988/10,700) in 2018. The opposite trend is visible among PWID. Each year between 1996 and 2004, PWID were the transmission group with the highest number of reactive tests. Since 2005, the proportion of this group has gradually diminished, reaching the lowest value (0.7%; 71/10,700) in 2018. In the same period, the proportion of all reactive tests for MSM (MSM plus MSW) increased, accounting for 95.0% of the total number of reactive tests detected in 2018.

**Figure 4:**
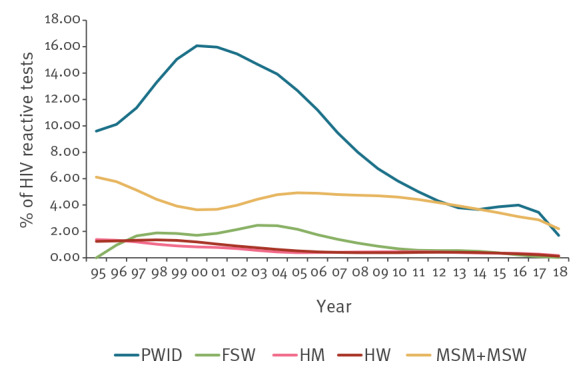
Percentage of HIV reactive tests by transmission group per year, Catalonia, Spain, 1995–2018 (n people tested = 106,992^a^; n reactive tests = 2,848^b^)

DEVO: voluntary detection; FSW: female sex worker; HM: heterosexual men; HW: heterosexual women; MSM: men who have sex with men; MSW: male sex worker; PWID: people who inject drugs.

^a^ Only people tested within the DEVO network were included. Before 2014 disaggregated data refers to number of tests instead of people tested.

^b^ Only HIV reactive tests performed within the DEVO network were included.

The highest reactivity rate in each transmission group during the whole period of study was found in PWID (ranging between 1.1% (1/95) in 2014 and 25.0% (8/32) in 2000), followed by MSM plus MSW (ranging between 2.2% (181/8,209) in 2018 and 10.2% (17/167) in 1995) (Figure 4). Nevertheless, in recent years the reactivity rate in the PWID group has gradually decreased. This decrease is not statistically significant, due to the low number of PWID tested. The reactivity rate in MSM plus MSW has shown a statistically significant decrease (p < 0.001), especially in the past 10 years, reaching 2.2% (181/8,209) in 2018. For the rest of the groups, no significant trend was observed.

The lines were smoothed using the moving average method.

**Figure 5:**
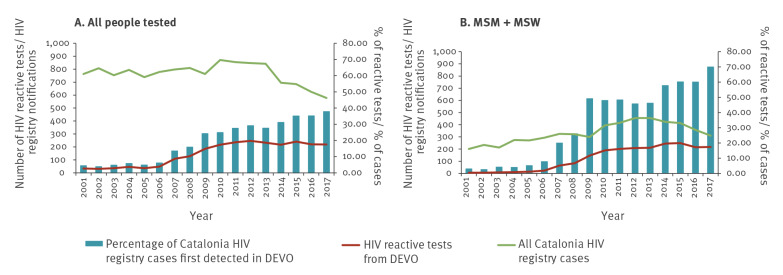
Number of HIV reactive screening tests from the DEVO network, total number of cases registered in the Catalonia HIV registry and the percentage^a^ of tests recorded in the Catalonia HIV registry first detected in the DEVO network for (A) all people tested (n = 2,483 in DEVO network, n = 13,004 in HIV registry) and (B) men who have sex with men and male sex workers only (n = 2,099 in DEVO network, n = 5,660 in HIV registry), Catalonia, Spain, 2001–2017

DEVO: voluntary detection; MSM: men who have sex with men; MSW: male sex worker.

^a^ Percentage calculated as number of reactive tests obtained by DEVO community testing divided by number of total cases recorded in the Catalonia HIV registry.

Figure 5 shows the increase of the contribution of HIV POCT in the community to the overall total number of cases registered in the Catalonia HIV registry. The percentage of positive cases in the Catalonia HIV registry which were first detected in the DEVO network has gradually increased, from 4.5% (34/763) in 2001 to 37.9% (219/578) in 2017. In the case of MSM plus MSW, this contribution is higher, reaching 70.0% of total HIV diagnosed cases in the Catalonia HIV registry among MSM in 2018.

In 2018, a total of 14,537 tests were performed in the DEVO network on 10,894 individuals, of which 1.8% (191/10,894) were reactive. Of these reactive tests, 94.8% (181/191) had a confirmatory test, of which 100% were confirmed as positive. Of the total number confirmed positive, 93.9% (170/181) were linked to care. The percentage of individuals with a reactive screening test who were linked to care was 89.0% (170/191). This percentage has not varied considerably since these data were available (81.7% in 2014, 95.0% in 2015, 89.5% in 2016, and 92.7% in 2017).

## Discussion

This study shows the contribution of community-based HIV POCT in improving early HIV diagnosis in Catalonia over time, especially among key populations, and demonstrates that the collected data are an important source of strategic information to be included into the Integrated AIDS/HIV/STI Surveillance System of Catalonia (SIVES).

In Catalonia, community-based HIV testing has been monitored and has formed part of HIV Surveillance since 1995. The DEVO Network has made it possible to collect standardised data on each person tested in CBVCT services. The collected data complement strategic information on key populations and thus make it possible to improve HIV prevention strategies aimed at these key populations. The continual monitoring performed by the DEVO Network has improved public health decision-making at the Public Health Agency of Catalonia by detecting changes in HIV testing uptake, in HIV tester profiles and in HIV test-seeking behaviours [5].

The DEVO Network succeeded in scaling up HIV testing among key populations, with the number of tests performed in the year following the implementation of POCT increasing by 103% from the previous year [5].

HIV POCT have the potential to increase the number of people who know their HIV status [24]. The POCT that meet the WHO’s ASSURED (affordability, sensitivity, specificity, user-friendly, rapid and robust, equipment-free and deliverable) criteria [25] follow a simple procedure involving a limited number of steps and are equipment-free, ensuring they can be performed outside traditional laboratory settings by staff with no formal laboratory training [24]. Additionally, both providers and clients prefer rapid tests over traditional tests [26,27]. Several studies have shown the efficacy of CBVCT strategies using HIV POCT to improve HIV testing uptake in populations at higher risk of exposure to HIV [6,7,9]. The DEVO Network has shown to be successful in providing testing to at-risk populations. In the period 1995–2018, 57.2% of tests were performed on MSM, 7.6% on SW (FSW plus MSW), 2.6% on PWID, 1.5% on transgender people and 45.2% on foreign nationals (including migrant population). A recent study showed that in Catalonia, 12.3% of those living with HIV were still undiagnosed, and this proportion was higher in migrants [28]. Therefore, in Catalonia, providing access to HIV POCT in the community is important, especially for populations facing barriers to accessing the healthcare system, such as the migrant population.

The low number of PWID tested in the DEVO network can be explained by the fact that most PWID are tested in harm reduction centres. In Catalonia there is a network of harm reduction programmes run by mobile units, street teams or facility-based centres. The facility-based centres are located in areas of drug trafficking and drug consumption, or in drug treatment clinics [29].

Linkage to care and treatment for those with a reactive test in the DEVO network is high (89%). A recent systematic review and meta-analysis of studies in the WHO European Region [30] showed a pooled estimate of 85% (95% CI: 75–93) of people with reactive tests linked to care within 3 months. Linkage of those with a reactive test to appropriate specialist services is a key step in the HIV continuum of care, as immediate initiation of treatment has substantial benefits in reducing the risk of patient morbidity, as well as reducing onward transmission [30].

In the past 10 years, a statistically significant decreasing trend has been observed in the percentage of MSM with a HIV reactive test. This trend could be explained by the success of different strategies of combined prevention in this key population, including increased testing frequency and earlier initiation of HIV treatment. BCN Checkpoint (the CBVCT service with the largest HIV testing activity, particularly among MSM) has gone further to promote earlier initiation of HIV treatment by introducing qualitative PCR POCT for the detection of acute HIV infection [31]. This, coupled with their pre-exposure prophylaxis (PrEP) service in the framework of research studies, has broadened the portfolio of preventive services available to users of the Checkpoint. In England, the incidence of new HIV infections in MSM attending sexual health clinics fell by 55% in 2016 and 2017 [32,33], and was attributed to an increase in HIV testing, earlier initiation of HIV treatment and the scale up of privately purchased generic PrEP in England from late 2017 onwards.

In Catalonia, universal treatment (treatment independent of CD4^+^cell count for patients newly diagnosed with HIV) has had a positive impact on the dynamics of the viral load in people living with HIV [34]. This, along with increasing testing and linkage to care as part of a combined prevention strategy, can explain the decrease in the percentage of new HIV diagnoses in the DEVO network. The increase in number of sites offering HIV testing thanks to the introduction of HIV POCT has increased the proportion of community detected HIV cases in the overall number of HIV cases reported in Catalonia, increasing from 4.5% in 2001 to 37.9% in 2017. The impact of introducing HIV POCT was even larger for MSM, where 70% of all new HIV diagnoses in 2017 were diagnosed in the community setting. This suggests that CBVCT services are a valuable element of the strategy to increase HIV testing in Catalonia, especially for MSM. These estimations are higher than that presented in a 2019 study showing that in several southern European countries, 0.2–19.7% of total HIV cases and 0.5–37.0% of HIV cases among MSM were diagnosed through CBVCT services [35].

The longstanding experience of the DEVO Network and its results has been used as a basis for establishing the COBATEST Network, a European network of CBVCT services that share standardised data [8,14].

There are a number of limitations to this study. Firstly, the disaggregated data presented in the Table from 1995 to 2013 refers to the number of tests performed, while data from 2014 to 2018 refer to the total number of people tested. This is due to improvements made to the data collection system and its integration into the COBATEST network. After 2013 a unique identifier was assigned to each client, allowing detection of repeat testers, and at the same time ensuring the anonymity of people tested. This could have led to an under-estimation of the number of tests in the period 2014–2018, especially in the MSM group as MSM are more often repeat testers. Secondly, the number of HIV positive cases from1995 to 2007 refers to HIV confirmed cases only, as the test offered was the conventional laboratory test. Since the introduction of HIV POCT, the number of HIV positive cases refers to reactive cases as in some cases, the information related to referral and confirmation of the diagnosis is not complete. So the number of HIV reactive cases in the period 1995–2006 could be higher. Lastly, regarding the number of cases detected in the DEVO network as a percentage of the total number of Catalonia HIV registry cases - as it was assumed that all reactive cases were linked to care and therefore were added to the Catalonia HIV registry, the contribution could have been overestimated.

### Conclusion

In conclusion, our study with a monitoring series of almost 25 years reinforces the important role that community-based HIV POCT has on the improvement of early HIV diagnoses in key populations, and highlights the importance of monitoring these data and including them in a regional or national HIV surveillance system. To ensure sustainability of the community testing services, key stakeholders must commit to including CBVCT services in the design and plan for strategies to achieve the 90–90–90 objectives.
